# Designing a physical activity parenting course: Parental views on recruitment, content and delivery

**DOI:** 10.1186/1471-2458-12-356

**Published:** 2012-07-05

**Authors:** Russell Jago, Joanna K Steeds, Georgina F Bentley, Simon J Sebire, Patricia J Lucas, Kenneth R Fox, Sarah Stewart-Brown, Katrina M Turner

**Affiliations:** 1 Centre for Exercise, Nutrition & Health Sciences, School for Policy Studies, University of Bristol, Bristol, UK; 2 Centre for Research in Health and Social Care, School for Policy Studies, University of Bristol, Bristol, UK; 3 Warwick Medical School, University of Warwick, Coventry, UK; 4 School of Social and Community Medicine, University of Bristol, Bristol, UK

**Keywords:** Parenting, Physical activity, TV, Intervention

## Abstract

**Background:**

Many children do not engage in sufficient levels of physical activity (PA) and spend too much time screen-viewing (SV). High levels of SV (e.g. watching TV, playing video games and surfing the internet) and low levels of PA have been associated with adverse health outcomes. Parenting courses may hold promise as an intervention medium to change children’s PA and SV. The current study was formative work conducted to design a new parenting programme to increase children’s PA and reduce their SV. Specifically, we focussed on interest in a course, desired content and delivery style, barriers and facilitators to participation and opinions on control group provision.

**Methods:**

In-depth telephone interviews were conducted with thirty two parents (29 female) of 6–8 year olds. Data were analysed thematically. An anonymous online survey was also completed by 750 parents of 6–8 year old children and descriptive statistics calculated.

**Results:**

Interview participants were interested in a parenting course because they wanted general parenting advice and ideas to help their children be physically active. Parents indicated that they would benefit from knowing how to quantify their child’s PA and SV levels. Parents wanted practical ideas of alternatives to SV. Most parents would be unable to attend unless childcare was provided. Schools were perceived to be a trusted source of information about parenting courses and the optimal recruitment location. In terms of delivery style, the majority of parents stated they would prefer a group-based approach that provided opportunities for peer learning and support with professional input. Survey participants reported the timing of classes and the provision of childcare were essential factors that would affect participation. In terms of designing an intervention, the most preferred control group option was the opportunity to attend the same course at a later date.

**Conclusions:**

Parents are interested in PA/SV parenting courses but the provision of child care is essential for attendance. Recruitment is likely to be facilitated via trusted sources. Parents want practical advice on how to overcome barriers and suggest advice is provided in a mutually supportive group experience with expert input.

## Background

Physical activity (PA) is associated with lower body mass, lipid and blood pressure levels among youth [1]. Screen-viewing (e.g. watching TV, playing video games and surfing the internet) has been associated with increased body mass, increased risk of metabolic syndrome and lower psychological well-being among children and adolescents [2-5]. A number of studies have reported that a considerable proportion of children spend more than two hours per day watching TV [6-8] and do not meet the current UK recommendation [9] of an hour of moderate-to-vigorous intensity PA (MVPA) every day [10]. Previous research has identified that the early primary school years (6–8 years of age) are a key period when children’s PA and screen-viewing behaviours are established [3] and as such encouraging active lifestyles at this age is likely to be critical.

Systematic reviews have indicated that there is a shortage of effective interventions to change children’s PA and screen-viewing (SV) [11,12]. The majority of interventions have been based in schools and have attempted to change behaviour via the curriculum. These school-based interventions have yielded either weak or inconclusive effects suggesting that alternative approaches are needed.

Parental facilitation of PA opportunities is associated with higher levels of PA among boys and girls [13-17]. Similarly, parental monitoring of SV and SV rules are associated with lower levels of SV among children and adolescents [18,19]. These data suggest that parents are important influences on children’s PA and SV but there is a lack of research on strategies to increase the effectiveness of parents in increasing PA and reducing SV among their children. One approach is to offer parenting courses. Parenting courses have proved highly successful in helping parents to improve anti-social behaviour, treat and prevent childhood obesity and are also widely used to reduce substance abuse but are as yet untested in promoting PA and reducing SV in children [20-25]. Given the lack of research in the area, it is not clear how to optimise the design of a PA and SV parenting course.

Optimising the design of complex interventions including content, participant recruitment and retention strategies is central to a phased approach to intervention development and testing [26]. Integral to this development stage is the engagement of participants in the planning process who reflect the intended user group (in this case parents of children aged 6–8 years) to elicit their opinions and perspectives. These perspectives can then be used to address logistical (e.g., location, frequency, duration), strategic (i.e., recruitment and retention strategies) and content aspects of the intervention so they are more likely to be attractive and acceptable to the target population. This paper reports the results of formative research conducted to inform the development and design of a PA and SV parenting programme. Specifically we sought to: 1) identify factors that might affect parent recruitment and participation; and 2) seek input from parents on course content and structure.

## Methods

Data were collected via in-depth interviews with parents and an anonymous online survey that was distributed via a national parenting website. The interviews were conducted first (January to February 2011) and the survey six months later (September – October 2011).

### Interviews

Parents were recruited via face-to-face contact with parents attending after-school activities, local community events and letters sent home to parents from local primary schools in two neighbouring wards in Bristol, UK. The wards are in the lowest and middle tertiles of deprivation according to the multiple index of deprivation (IMD)[27] for the city of Bristol and are therefore deemed to approximate low and middle socioeconomic status (SES) areas of the city. We intended to recruit an equal sample from each area with a spread of males and females. The final sample was comprised of thirty two parents (29 female, 3 male) with 17 from the low SES area and 15 from the middle SES area. All interviews were conducted between January and February 2011. All participants had at least one child aged between 6 and 8 and an average of 2.2 children. A guide was used to ensure consistency across the interviews and the semi-structured format allowed participants to raise pertinent issues. The guide focused on logistical questions around recruitment and retention (e.g. “how many classes do you think would be acceptable to attend?”, “how long should the course last?”, “how often should they be?”, “what time of day would suit you?” and “how long do you think each session should last?”) what should be included in the sessions in terms of topics and activities.

All interviews were conducted by telephone (mean length 20 minutes and 24 seconds). Telephone interviews were chosen as it is more convenient for parents and studies have shown that participants are more likely to answer potentially sensitive questions over the telephone [28-31]_._ All interviews were digitally recorded and transcribed verbatim.

### Interview analysis

Data were analysed thematically [32] so that comparisons could be made within and across the interviews. The approach involved researchers reading and re-reading the interview transcripts in order to identify themes and to develop a coding frame. Transcripts were coded independently by several members of the research team so the coding frame could be refined through discussion. Researchers met to discuss areas of consensus and discrepancy. This led to further codes being developed and existing codes being defined more clearly. The coding frame was developed and revised by hand with a sample of transcripts and then all transcripts were entered into NVivo (Version 9.0, QSR, Southport, UK) to allow for electronic coding and retrieval of data. Once all transcripts had been coded, data were analysed by performing text retrievals on codes, contents were interpreted, summarised and indicative quotes that captured the essence of broader views identified for inclusion in this paper.

### Survey

The online survey was designed to explore the salience of the logistic issues that were raised in the interviews with a larger sample of participants. The survey also examined respondents’ opinions about potential control group provision and recruitment methods which would be important for designing a pilot trial evaluation of a PA parenting intervention. An advertisement inviting parents of 6–8 year old children to complete a short anonymous survey was placed onto the message boards of Netmums (a UK parenting website). Participants were asked to report age, gender, their current employment status and the highest level of education within the household. Measures assessing potential factors pertaining to recruitment were developed for this study. Parents were then asked to report the perceived importance (*very important, important, not important and not important at all*) of seven factors that might affect recruitment into a PA parenting course. Parents were also asked the extent to which they paid attention to (*always pay attention, often pay attention, rarely pay attention and never pay attention*) seven different means of communication of course information (e.g., about PA). Finally, parents were asked to indicate their thoughts about the perceived value (*great alternative, ok as an alternative, not very good and not good enough*) of four different options that could be provided for a control group who did not receive a PA parenting intervention. The survey analysis was descriptive with the number and percent of responses to each question was calculated and tabulated.

Both studies were approved by a University of Bristol ethics committee. Written informed consent was provided for all interviews. As the online survey was anonymous participants were informed that by consenting to take part in the survey that they were providing informed consent but written informed consent was not obtained.

## Results

### Interviews

Analysis yielded eight themes: 1) interest in a parenting course; 2) barriers to attending a parenting course; 3) facilitators of attending a parenting course; 4) formatting recruitment materials; 5) recruitment locations; 6) preferences for course content; 7) preferences for delivery style and 8) session frequency, length and duration.

### Interest in attending a PA parenting course

Although many parents from both areas said they were interested in and would go to a PA-based parenting course, this was reported more frequently by parents in the low SES area. Parents reported they were interested in the course because they wanted ideas and advice about PA, because they specifically wanted to help their child or because they felt it would help them with other areas of concern (e.g. parenting skills).

“Well we've got quite a big family erm I mean I'm open to pretty much any idea that would help them be more … because when they're well exercised and stuff they're calmer. And calmer kids is all good by me” (023 Mother, Low-SES)

"“Yeah, I would, I’d quite like learn things, to find out what is around and about that you can use.” (029 Mother, Low-SES)"

"“Yes, yeah, very interesting, yeah. Because if you’ve got a lot of, you know, children as mine, they are not doing enough, you know, PA and stuff.” (017 Father, Middle-SES)"

### Barriers to attending a parenting course

As the parenting course would be attended only by parents without their children the majority of parents considered childcare to be a barrier to attending a parenting course:

"“Just the childcare, that’s the only thing, barriers of who would look after the kids.” (002 Mother, Middle-SES)“"

"“..if it was after school and I’d have to bring all 4 of the kids, or. ..I’d have to sort out someone to look after my kids” (007 Mother, Middle- SES)"

For some parents, childcare would only present as a barrier if both parents were to attend.

"“But only if there was childcare provided (could we attend), because otherwise we’d be stuck where they’d only be one of us able to do it.” (028 Mother, Low-SES)"

Other barriers that parents reported were being busy, other commitments (including work) and financial cost for some.

"“the obstacles that you’ll probably come across with most parents is that they do stuff with their kids, i.e. swimming lessons …because when you’re trying to sort of arrange things with other parents it’s ‘oh no, we’ve got ballet tomorrow’.” (001 Mother, Middle-SES)"

"“…obviously we have got a lot of commitments in the evening … I think probably my only personal problem is whether I am free.” (016 Mother, Middle-SES)"

"“…because I’m looking into getting a part time job so for me it would depend on what I’m working and stuff.” (020 Mother, Low-SES)"

"“…money is quite an issue as well, with sort of, if you get the bus and everything, you’re talking £7.50 for me and [child’s name].” (022 Mother, Low-SES)"

### Facilitators to attending a parenting course

Common facilitators were offering free lunch/refreshments and the social aspect of attending a parenting course. In particular, social support and making new friends were noted by parents.

"“..obviously if you make friends with someone at a group and they’re five minutes down the road you could both meet up with your kids and go off and do stuff. (018 Mother, Low-SES)"

"“I think it probably would be quite good to meet other people … I can’t be the only person that … feels like they need to do a little bit more.” (020 Mother, Low-SES)"

### Recruitment materials

Some parents mentioned that they would need to feel that the course would be appropriate for them by assessing their goals or needs. It would be important to address this in the promotional material.

"“I’d need to have some sort of, expectation… sort of aim or goal before …” (009 Mother, Middle-SES)"

"“…I think there is a danger that, you know that there … it’s just geared up towards, as I say, children who appear obese.” (015 Mother, Middle-SES)"

### Locations for advertising

The most commonly cited location for promotion of a parenting course was schools, possibly using face to face recruitment:

"“Yeah, but also you kind of always try and read everything from the school you don’t know… it might affect your kids.” (014 Mother, Middle-SES)"

"“Schools are fantastic, yeah schools are fantastic, because then it goes straight to the child, hopefully then if it goes through.” (027 Mother, Low-SES)"

"“Oh it is always the best way to go through the schools to get through to the parents…whereas half the time if you just send out leaflets and stuff and not see them face-to-face the chances of getting them is very difficult, whereas if they actually have contact with you and they feel at ease with you, then they don’t mind so much.” (011 Mother, Middle-SES)"

Other suggestions for promotion included children’s centres or nurseries, healthcare surgeries and local projects.

"“I know that I always look at the adverts in places like doctors’ surgeries, dentists and playgroups, cafes. Even like the local coffee shops that you have got around sort of areas, you know people pick up leaflets from there.” (016 Mother, Middle-SES)"

### Preferences for course content – learning approach and topics to be covered

Receiving advice was valued by most parents, and this took two forms: advice from experts and advice from other parents. The majority of parents reported that sharing ideas and learning from the experience of others would be important.

"“I suppose until I’ve really done one I wouldn’t really know but I just, I do like the idea of you know talking with other parents because they’re living it.” (001 Mother, Middle SES)"

"“You learn a lot from other parents so I would definitely value that” (003 Mother, Middle SES)"

"“I just think that you know, bouncing ideas off everybody, you know if it’s just me it’s not, you know you just hear my point of view, you’re not hearing all the other people, it’s always more heads are better than one.” (001 Mother, Middle-SES)"

"“I prefer a group, then you pick up on people’s ideas because I might not have thought of something you know” (019 Mother, Low-SES)"

The inclusion of professional or expert advice and workshops/demonstrations was also valued.

"“… like obviously the professionals… Because they’ve obviously been there, done it, got the t-shirt sort of thing.” (018 Mother, Low-SES)"

"“It’s obviously great to have the professional input, but to hear on a more practical level what has worked for others you know, it could well be something that you hadn’t thought of, something so sort of simple.” (027 Mother, Low-SES)"

Parents also talked about their preference for topics to cover in the course. Some parents described how they would benefit from knowing what would be appropriate types and quantities of PA for their children.

"“..it would be interesting to know what, you know, the experts think about well what’s the right amount of exercise…” (001 Mother, Middle-SES)"

"“So what things do you normally do that could actually qualify as exercise and how much more would you have to do to sort of take them over a boundary…” (003 Mother, Middle-SES)"

Most parents said that they would like more ideas of activities to do with their child with a particular interest in low costs ideas as well as ideas for being active in bad weather, limited space and limited time.

"“..some play thing that might be easier to do in a sort of small space and that’s not organised but can be done sort of in you know ten or twenty minutes just to keep you sort of ticking over” (004 Mother, Middle-SES)"

"“A sheet full of ideas and things that you can do, places that you can go that don’t cost money and where you kids can get some exercise” (028 Mother, Low-SES)"

Other parental suggestions included content on healthy eating, help with scheduling and planning PA and motivating their children to do more PA.

"“For me personally it would be more … making my son understanding how important it is. You know, us as parents, we spend a long time saying like, it’s good to do this. But instilling that into them would … would be good” (020 Mother, Low-SES)"

"“It would be nice to know how to plan, I have a major problem with planning ..” (023 Mother, Low SES)"

### Preferred delivery style

The majority of parents reported that they would prefer a group-based approach as it was perceived to provide opportunities for sharing ideas and learning from others.

"“I’m not normally a group working person, but this sort of thing you know, where you can sort of share ideas and, well even if you haven’t got any view, just to be able to sort of learn from others.” (027 Mother, Low-SES)"

Many parents talked about benefiting from the social support offered through a group. In addition, parents frequently cited feeling more comfortable in a group session and feeling less isolated.

"“If you make friends with someone at a group and they’re five minutes down the road you could both meet up with your kids and go off and do stuff……And obviously if you’re, if you’re, if you are finding it really tough obviously to get the kids out then you’ve got the emotional support there from other parents….” (018 Mother, Low-SES)"

Making new friends and meeting new people were important aspects of the group approach for some parents.

"“..it is an easy way to meet other people as well.”(007 Mother, Middle-SES)"

### Session frequency, length, duration and timing

In response to the question about the desired course duration the participants indicated that four sessions was preferred because this number seemed achievable for families, but was also long enough for parents to feel involved:

"“No, I think that (4–5 sessions) would be perfect, that’s enough to keep them interested but you know not enough to sort of you know where it’s too much.” (021 Mother, Low-SES)"

Parents gave similar rationales for their preferences for weekly sessions of about 90 minutes. A regular slot is more likely to be remembered:

"“I think weekly, personally I’ve found weekly is a big commitment but you get more benefit, if it’s two or three weeks sometimes people forget, you know.”(005 Father, Middle-SES)"

"“Yes I think it should so people get into the habit, oh every Thursday this is going to be on for the next five weeks…..” (006 Mother, Middle-SES)"

An hour and a half was felt to allow time to settle in, and to discuss material in depth:

"“Because when people arrive they tend to sort of be fidgeting around and sitting and maybe chatting with other people, so you know, you need that. Whenever I go to things they’re always like an hour and a quarter because there’s always that little bit at the beginning…” (001 Mum, Middle-SES)"

"“No I think that’s quite good [90 minutes] because once you get in, everyone says I'm here that’s 10 minutes gone isn't it?” (023 Mother, Low-SES)"

A small number of parents, all in the Middle SES area, said they would prefer more than 5 sessions, one commented that this was because child care commitment would be difficult to alter for a short period of time.

"“….there’s nothing worse, you can’t really alter your child care commitments if it’s just for about four weeks …” (003 Mother, Middle-SES)"

Parents in both locations gave similar preferences to the time of the day of the session, and in all cases these were related to practical difficulties of attending rather than affective factors. Most parents said that the daytime would be preferable, and a smaller number said that the evening would be most suitable. Daytimes were felt to fit better around flexible work patterns and because school aged children would be at school.

"“Once children are in school you’ve got that school time” (010 Father, Middle-SES)"

Parents who regarded evenings to be most suitable, explained their preference due to work commitments in the day or because they would be looking after young children in the day.

"“Because I work it would be evening definitely.” (002 Mother, Middle-SES)"

Overall it is noticeable that the interviews indicate that the parents were very much focussed on the logistical issues associated with attending a physical activity parenting programme with less focus on how to actually increase children’s physical activity*.*

### Quantitative data

The survey was live for a total of 4 weeks and 750 responses were recorded. Demographic characteristics of the survey respondents are presented in Table 1. The sample was predominately female (97.5%) and most were working full or part time (63%) and had stayed in education at least through further education (97.5%). The age ranged between 19 and 57 years of age (mean =35.5, SD = 6.0).

**Table 1:** Demographic characteristics of survey respondents (n = 750)

	**Mean**	**SD**
**Age (years)**	35.5	6.0
**Gender**	**N**	**%**
Male	8	1.1
Female	731	97.5
**Missing**	11	1.5
**Current employment status**		
Full-time employed	157	20.9
Part-time employed	316	42.1
Housewife/husband	231	30.8
Student	23	3.1
Unemployed	23	3.1
**Highest education in household**		
Did not complete secondary school	19	2.5
GCSE or GNVQ^1,2^	177	23.6
A Levels/Advanced GNVQ	213	28.4
University degree	212	28.3
Postgraduate degree	129	17.2

^1^GCSE = General Certificate of Secondary Education, GNVQ = General National Vocational Qualification.

^2^ Both GCSE and GNCQ are normally obtained at age 16.

The frequencies of responses on the perceived importance of factors that might affect recruitment into a parenting course are presented in Table 2. In common with the qualitative data, practical constraints to attending were most important to parents. The single most important factors for parents was the timing of classes, with 89% of the sample reporting that this was very important. Over half of the respondents perceived that the time and dates of classes were very important when deciding whether to take part in a course. The provision of childcare was important to 62.9% of participants. The content of classes mattered to most parents with 56.9% reporting it was very important. Most parents were not concerned about who else would attend the group (75.5% not/not important at all) but rather with who would lead it (62.5% not/not important at all).

**Table 2:** Frequencies of responses on importance of factors affecting recruitment to physical activity parenting programme (n = 750)

	**Very important**		**Important**		**Not important**		**Not important at all**	
	N	%	N	%	N	%	N	%
**Time of classes**	665	88.7	80	10.7	4	0.5	1	0.1
**Dates of classes**	399	53.2	276	36.8	69	9.2	6	0.8
**Knowing who will be leading the classes**	116	15.5	358	47.7	251	33.5	25	3.3
**Description of classes**	427	56.9	313	41.7	9	1.2	1	0.1
**Who else will be in the group**	37	4.9	147	19.6	480	64.0	86	11.5
**Who the classes are organised by**	104	13.9	370	49.3	243	32.4	33	4.4
**Childcare provision**	225	30.0	247	32.9	214	28.5	64	8.5

The frequency of responses on the extent to which parents might pay attention to different recruitment methods are presented in Table 3. Over 90% of parents reported that they always pay attention to a letter home from school while over 80% of parents reported that they always or often pay attention to someone at school. Similarly, over 90% of parents reported that they always or often pay attention to “word of mouth”.

**Table 3:** Frequencies of responses on importance of salience of recruitment methods

	**Always pay attention**		**Often pay attention**		**Rarely pay attention**		**Never pay attention**	
	**N**	**%**	**N**	**%**	**N**	**%**	**N**	**%**
School – letter home	686	91.5	57	7.6	3	0.4	4	0.5
School – information on a board	84	11.2	342	45.6	289	38.5	35	4.7
Someone at school (i.e. teacher or assistant)	250	33.3	386	51.5	105	14.0	9	1.2
Posters/flyers in local places (i.e. church hall, sport centre)	73	9.7	389	51.9	272	36.3	16	2.1
Health professional (i.e. nurse, community worker)	112	14.9	291	38.8	283	37.7	64	8.5
Advert/feature in local paper	88	11.7	371	49.5	254	33.9	37	4.9
Word of mouth	268	35.7	436	58.1	41	5.5	5	0.7

The perceived value of possible options for participants assigned to a control group within a controlled trial of a PA parenting course are presented in Table 4. The most preferred option was the opportunity to attend the same course at a later date followed by a voucher for a local sports class. The two sources of data had good agreement, parents reported similar factors as important in both interviews and in an online questionnaire. A summary of the key findings from the two sources of data and the implications of those findings for the development of a PA/SV parenting intervention is presented in Table 5.

**Table 4:** Frequencies of responses on the value of control group provision for a parenting intervention

	**Great alternative**		**OK as an alternative**		**Not very good**		**Not good enough**	
	**N**	**%**	**N**	**%**	**N**	**%**	**N**	**%**
There was a chance to attend the same programme at a later date	441	58.8	296	39.5	11	1.5	2	0.3
At the end of the programme there was the chance to attend 1 day programme instead	173	23.1	447	59.6	118	15.7	12	1.6
At the end of the study all of the materials were provided but there were no classes	114	15.2	309	41.2	277	36.9	50	6.7
At the end of the study a voucher for local sport classes was provided	295	39.3	319	42.5	106	14.1	30	4.0

**Table 5:** Implications of the user-group engagement and developmental research findings for the design of a PA/SV parenting intervention

**Developmental research findings**	**Intervention design implications**
**Facilitators and barriers** Childcare needs Timing - not afterschool - finding free time to attend Costs of travel to intervention sessions Social aspects of course - desire for group sessions - meeting friends on course - emotional support from group	Provision of free child care for parents attending intervention sessions Participants given option of attending daytime course while children are at school or evening course. Intervention delivered in multiple locations close to sampling area to minimise the need to travel Group-based course Focus on sharing experiences
**Recruitment** Programme expectations/marketing/description of sessions Time of sessions Location/methods - Schools - Known contact at child’s school - Face-to-face at schools - Coffee shops, leafleting etc. - Word of mouth	Recruitment materials (i.e., leaflets, posters, website, online advertising) designed to include key information reported to influence decision to enrol and aligned with the desired content as outlined below (e.g., social/group sessions, key topics covered and length of sessions). Varied and wide-reaching recruitment campaign including; letters to parents via schools, coffee mornings at schools and children’s centres, attendance at afterschool activities, family events, parent-toddler groups, stall at school fetes, meeting parents directly at schools, liaison with key community members, study website, posters and fliers in local venues and press releases.
**Intervention Content & learning methods** **-** what “counts” as exercise - ideas for active play/unstructured activity - ideas of where to go for free to be active - planning for PA - communicating about activity with children - encouraging child’s motivation	All desired content was incorporated in to the sessions. (e.g., Session 1: what counts as PA; Session 2: active play; Session 3: communication; Session 4 encouraging motivation; Session 7: Planning for PA - Dedicated time for parent feedback on parenting techniques attempted in the previous week - Significant time dedicated to practical advice. Take home guides on “putting it into practice” - Time given each week for parents to talk about their own experiences to support peer learning.
**Learning methods** - learning from other parents - professional advice - practical advice	- Development of an Activity Directory providing ideas of physical activity opportunities

## Discussion

The two independent sources of data that were collected in this study indicate that parents would be interested in attending a PA/SV parenting course but logistical issues may hinder attendance. Key issues were the provision of childcare, timing of sessions and balancing other time commitments that were either work- or family-based such as the needs of other children in the family. These findings are consistent with the participant retention strategies (e.g., provision of instrumental supports such as child care) identified by researchers conducting health behaviour change interventions [33]. Collectively, these findings imply that a PA parenting course may hold promise as an intervention medium but such an intervention can only work if the sessions are scheduled at a time to meet the needs of the participants and for many parents the provision of childcare is critical to attending a parenting course.

Recruitment is critical to the success of any course and the wide range of recruitment approaches reported in parenting interventions and parenting focussed obesity prevention interventions [34-39] indicate the difficulties associated with parental recruitment. While addressing the logistical issues outlined above may facilitate recruitment this is still likely to be a critical task for those providing of parenting courses. The data presented in this paper indicates that schools and school staff were highly trusted sources of information and the perceived optimal delivery mechanism. The survey respondents also indicated that information that was shared via “word of mouth” in the local community was perceived to be highly trusted. This finding is consistent with previous local research which has reported that information from friends and peer groups is highly trusted and likely to be an effective means of recruiting adults into community based PA courses [40].

Parents who took part in the interviews reported the key issues they would like to be addressed in a PA parenting course were an understanding of the recommended levels of PA, ideas for low cost activities, wet weather activities and how to structure days to allow time for PA. These issues map onto the barriers of lack of time knowledge, money and PA opportunities that have been consistently reported by children and adults as barriers to PA participation [31,40,41]. The data presented here therefore provide guidance on the key content that needs to be included into a PA and SV parenting course to help parents facilitate behaviour change for their children. As noted in the results, it is however noticeable that the parents focussed on logistical issues and did not focus on how to motivate children to be physically active. The focus on logistical issues may reflect the knowledge of the parents as it would be difficult for parents to comment on how to motivate change in behaviour if they have not previously participated in a PA/SV parenting intervention.

In terms of delivery style, the interview participants reported that they wanted an opportunity to share experiences with other parents but highly valued expert opinion. The parents also reported that weekly sessions would enable them to build their skills. As such the parents were advocating content that is consistent with general parenting courses. For example both the Nurturing Programme [42] and the Incredible Years Programme [43] are group based parenting courses that include weekly sessions in which parents share experiences in a safe and supportive environment. Combining this delivery style with expert content on recommended levels of PA and SV and how to change behaviour may therefore form the basis for a new PA and SV parenting intervention.

Respondents expressed a preference for a delayed parenting programme for the control group further reinforcing that a PA/SV parenting programme is likely to be valued by parents. “Wait list” control groups have been used in a number of studies and can be advantageous for recruitment as both intervention and control group participants perceive that they are receiving a benefit from taking part in the study. Wait-list control groups do, however, limit the ability to examine the longer-term effect of an intervention. As such, while a wait-list control group as intrinsic appeal to researchers and participants its application to a trial setting may be limited.

### Strengths and limitations

The major strength of this study is the provision of new information on the factors that would affect recruitment into a PA parenting course, retention once enrolled and input on course content from both a qualitative and quantitative perspective. The study does, however have a number of limitations that need to be acknowledged. Firstly, only 32 parents were included in the interviews which although comparable with similar qualitative studies [40] means we cannot generalise our findings to other groups and settings. Equally, both the qualitative and quantitative samples included very few fathers and therefore we are limited in our ability to comment on the views of these groups. Secondly, the quantitative data were collected from a parenting website via an anonymous survey. As such it is likely that respondents had a heightened interest in seeking guidance on parenting issues. Finally, we did not collect data on PA levels of parents or children so we cannot draw any conclusions about the current activity levels of this group of parents and therefore whether they represent the views of a group likely to benefit from a PA/SV parenting course.

## Conclusions

The data presented indicate there is an interest in parenting courses that focus on facilitating increased PA and reduced SV for children but key logistical issues, particularly the provision of child care needs to be overcome to enable parents to attend. The data also indicate that recruitment to parenting courses is likely to be facilitated via trusted school and friendship sources. In terms of content, parents want practical advice on how to overcome barriers and suggest that this could be facilitated via a mixture of a mutually supportive group experience and expert input.

## Abbreviations

PA: Physical activity; SV: Screen-viewing.

## Competing interests

We have no conflicts of interest to declare.

## Author contributions

The study was conceived by RJ, SJS, PJL, KRF, SSB and KMT. The data were collected and analysed by JKS and GFB under the supervision of RJ and KMT. The first draft of the paper was written by RJ and all authors provided critical revisions for intellectual content. All authors have approved the final manuscript.

## Pre-publication history

The pre-publication history for this paper can be accessed here:

http://www.biomedcentral.com/1471-2458/12/356/prepub
